# Efficacy and safety of programmed cell-death-protein-1 and its ligand inhibitors in pretreated patients with epidermal growth-factor receptor-mutated or anaplastic lymphoma kinase-translocated lung adenocarcinoma

**DOI:** 10.1097/MD.0000000000018726

**Published:** 2020-01-17

**Authors:** Olivier Bylicki, Florian Guisier, Isabelle Monnet, Hélène Doubre, Radj Gervais, Henri Janicot, Maurice Perol, Pierre Fournel, Régine Lamy, Jean-Bernard Auliac, Christos Chouaid

**Affiliations:** a Service de Pneumologie, Hôpital d’Instruction des Armées Percy, Clamart; b Service de Pneumologie, Centre Hospitalier Universitaire de Rouen, Rouen; c Service de Pneumologie, Centre Hospitalier Intercommunal de Créteil, Créteil; d Service de Pneumologie, Hôpital Foch, Suresnes; e Département d’oncologie, Centre François Baclesse, Caen; f Service de Pneumologie, Centre Hospitalier Universitaire de Clermont-Ferrand, Clermont-Ferrand; g Service d’Oncologie Thoracique, Centre Léon Bérard, Lyon; h Département d’oncologie, Institut de Cancérologie de la Loire, Saint-Priest-en-Jarez; i Service de Pneumologie, Centre Hospitalier Bretagne Sud-Lorient, Lorient; j Service de Pneumologie, Centre Hospitalier F. Quesnay, Mantes-la-Jolie, France.

**Keywords:** anaplastic lymphoma kinase translocation, epidermal growth-factor receptor-activating mutations, immune-checkpoint inhibitors, non-small–cell lung cancer, programmed cell-death-protein-1 and its ligand inhibitors, c-ros oncogene 1 translocation

## Abstract

Immune-checkpoint inhibitor (ICI) efficacy in patients with non-small cell lung cancer (NSCLC) harboring molecular alterations remains poorly elucidated. This study was undertaken to determine ICI efficacy against epidermal growth-factor receptor (*EGFR*)*/*anaplastic lymphoma kinase (*ALK*)*/*c-ros oncogene 1 (*ROS1*)-mutated NSCLC patients in the real-world setting.

In this retrospective, multicenter study on adults with ICI-treated *EGFR-*mutated or *ALK-* or *ROS1*-translated NSCLCs, we analyzed clinical characteristics and outcomes: ICI-treatment duration, and progression-free survival (PFS), objective response rate, duration of response, and overall survival (OS) from immunotherapy initiation.

Fifty-one NSCLC patients (mean age, 58.0 years) were included from 20 French centers: 61% were never-smokers and 59% were women. Among them, 82% had *EGFR*-activating mutations, 16% *ALK* translocations, or 2% *ROS1* translocations. Before ICI therapy, patients had received a median of 3 treatment lines (including tyrosine-kinase inhibitor). The median PFS was 2.1 (95% confidence interval [CI], 1.5–3.2) months for the entire cohort, 2.2 (95% CI, 1.4–3.2) for *EGFR*-mutated patients, and 2.4 (95% CI, 2.1–not reached) months for *ALK*-translocated patients. The median OS was 14.7 (95% CI, 12.1–19.2) months for the entire population and 13.9 (95% CI, 8.8–20.0) and 19.2 (95% CI, 13.1–not reached) months for *EGFR*-mutated and *ALK*-translocated patients, respectively. Seven (13.7%) patients were treated with ICI for >9 months. Toxicities were reported in 22% (11/51), including 8% (4/51) grade ≥3.

In this real-world setting, analysis of ICI PFS against *EGFR*-mutated or *ALK*-translocated NSCLC patients appeared close to that observed in pretreated unselected NSCLC patients. The more promising OS probably linked to post-ICI treatments. Large prospective studies on these patient subsets are needed.

**Key Points**

- Our results do not support decreased ICI efficacy in patients with *EGFR*-mutated or *ALK*-translocated NSCLC.
- In the real-world setting, ICI impact on *EGFR*-mutated or *ALK*-translocated unselected NSCLC was close to that previously observed but it should be used preferably after the failure of other therapeutics (tyrosine-kinase inhibitors and chemotherapy).
- Large prospective studies are needed to better define the place of ICI in the armamentarium for patients with *EGFR*-mutated or *ALK*-translocated NSCLC.

## 1 Introduction

The understanding of the molecular characteristics of tumor cells in non-small cell lung cancer (NSCLC) has changed considerably within the last decade.^[1]^ As a consequence, the management of patients with locally advanced or metastatic NSCLCs has been improved with innovative therapies, such as immune-checkpoint inhibitors (ICIs) and, for patients with oncogenic drivers, targeted tyrosine-kinase inhibitors (TKIs).^[2]^

Therapies targeting epidermal growth-factor–receptor (*EGFR*)-activating mutations were shown to be beneficial for patients harboring them. Notably, the authors of several phase III trials comparing EGFR-TKIs (gefitinib, erlotinib, afatinib) reported longer progression-free survival (PFS) and higher objective response rates (ORRs) compared with chemotherapy.^[3–11]^ However, despite these innovative therapies, patients finally progressed after a median of 9 to 12 months.^[12,13]^ Patients who acquire the *T790M* resistance mutation are eligible to receive a third-generation EGFR-TKI (e.g., osimertinib).^[14]^ For patients with *ALK* or *ROS* translocations, PFS increased under first-line crizotinib, compared with platinum-based doublet chemotherapy.^[15]^ Other TKIs that target translocated *ALK* have been developed to counter acquired resistance to crizotinib.^[16–18]^

Humanized monoclonal antibodies have been designed to block the interaction between programmed cell-death-protein-1 (PD-1) and its ligand (PD-L1) that is a negative regulator of T-cell anti-tumor defense.^[19]^ Both anti-PD-1 (nivolumab, pembrolizumab) and anti-PD-L1 (atezolizumab) ICIs have demonstrated their benefit in comparison with chemotherapy.^[20–25]^ Only low percentages of patients with *EGFR* mutations or *ALK* translocations were included in those trials. A meta-analysis showed no evidence of an advantage of second-line PD-1/PD-L1 inhibitors over docetaxel for patients with *EGFR*-mutated advanced NSCLCs.^[26]^ However, the small sizes of these subgroups and a posteriori analyses prevented drawing firm conclusions. Overall, about 200 patients with *EGFR* mutations and 20 with *ALK* translocations included in those randomized trials were treated with second/third-line PD-1/PD-L1 inhibitors.^[27]^

The purpose of this retrospective study in the real-world setting is to gain better understanding of *EGFR*-mutated or *ALK-* or *ROS*-translocated advanced NSCLCs treated with ICI after progression on targeted treatment.

## 2 Materials and methods

### 2.1 Study design and patients

The IMAD study (GFPC 03–2016) was a retrospective, multicenter study conducted in French Lung Cancer Group (GFPC) centers. Its primary objective was to assess ICI efficacy (ORR, duration of response [DOR], PFS, and overall survival [OS]) after progression on targeted therapy for NSCLCs harboring *EGFR* mutations or *ALK/ROS1* translocations. The secondary objective was the assessment of safety.

Adult NSCLC patients were enrolled in the study when they met the following criteria: lung adenocarcinoma with *EGFR-*activating mutations, *ALK* translocations, or *ROS1* translocations; prior targeted treatment for *EGFR* mutation or *ALK* translocation; ICI as second-or-more treatment line. Patients included in a clinical immunotherapy trial were excluded.

### 2.2 Data collection

Patient demographics and clinical characteristics at NSCLC diagnosis were obtained from patient files and included: age; sex; smoker status; ethnicity; cancer stage; number and sites of metastases; presence of *EGFR*-activating mutations, *ALK* translocations and *ROS1* translocations; treatment lines (chemotherapy or TKIs) before ICI; the Eastern Cooperative Oncology Group performance status (ECOG PS) at immunotherapy onset; clinical response to ICI therapy; adverse event (AE) type and grade on ICI; and post-immunotherapy treatment.

### 2.3 Statistical analyses

OPFS was defined as the time from ICI initiation to progression on ICI. Progression was defined as Response Evaluation Criteria In Solid Tumors version 1.1 criteria (RECIST 1.1)^[28]^ radiological or clinical progression (deteriorated clinical status preventing systemic treatment) or death. Assessments were done in each participating center without centralized imaging review.

OS was calculated from ICI starting to death, the ORR to ICI as the best observed according to RECIST1.1 (radiological assessment were done every 6 weeks). AEs were reported according to Common Terminology Criteria for Adverse Events (CTCAEs) version 4.

The Kaplan–Meier method was used to estimate PFS and OS for the entire cohort and according to the molecular genotypes.

All statistical analyses were computed with the RStudio statistical software (Version 1.1.383, RStudio, Boston, MA).

### 2.4 Ethical considerations

The study was conducted in accordance with the Declaration of Helsinki. Participating centers were responsible for obtaining patient consent and institutional approval. All contributors were trained in good clinical practices. The study was purely an academic collaboration and was not funded by industry.

## 3 Results

### 3.1 Patient characteristics

Fifty-one patients were included in 20 medical centers (Table 1). The mean (±standard deviation) age at diagnosis was 58.0 ± 8.8 years, 30/51 (59%) patients were women and 31/51 (61%) were never-smokers. They had a median of 3.6 (range, 1–7) metastatic sites at diagnosis. At that time, 42/51 (82%) patients had an *EGFR* mutation, 8/51 (16%) harbored an *ALK* translocation, and 1/51 (2%) carried a *ROS1* translocation. The most frequent *EGFR* mutations at diagnosis were deletion in exon 19 and point mutation in exon 21 (*L858R*), which accounted for 81% (34/42) of all *EGFR* mutations.

**Table 1:**
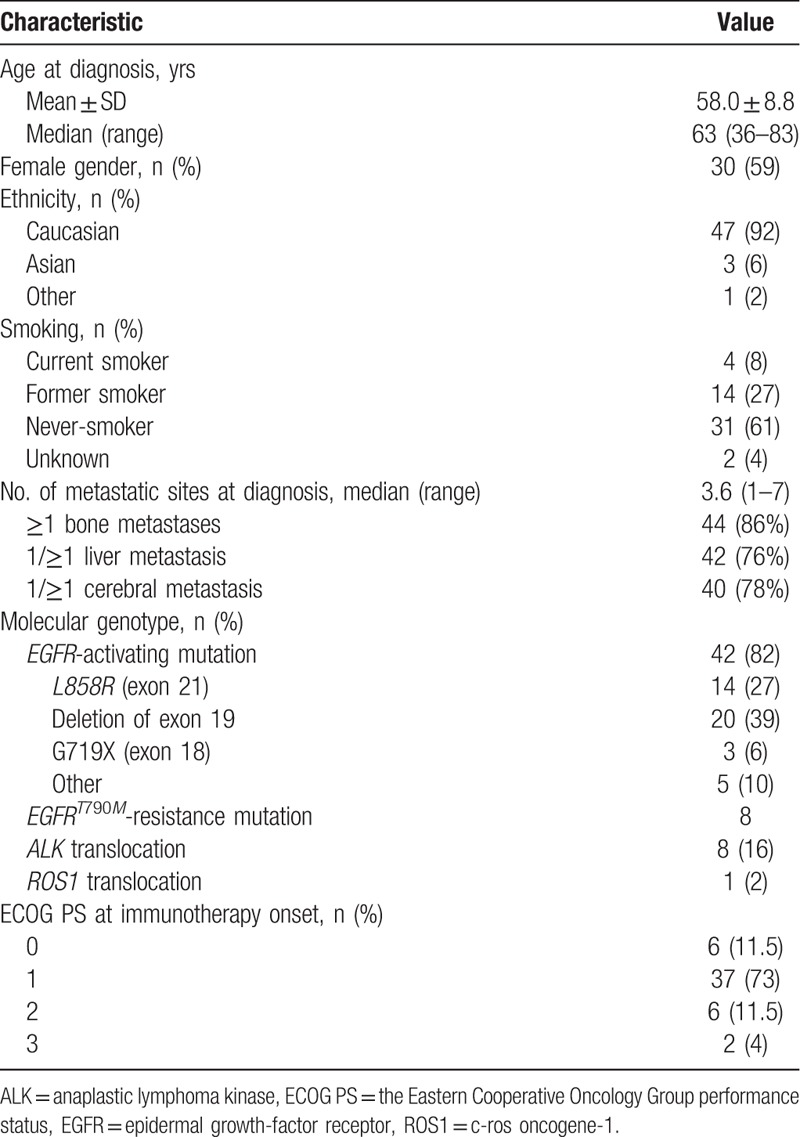
Characteristics of the 51 patients.

Before starting ICI therapy, patients had received a median of 3 (range, 1–9) treatment lines, including TKI for all patients: first-line treatment for 45% (23/51) and second-line treatment for 49% (25/51) (Table 2); 8/42 (19%) EGFR patients carried the *T790M* resistance mutation and received osimertinib as second- or third-line therapy before ICI introduction.

**Table 2:**
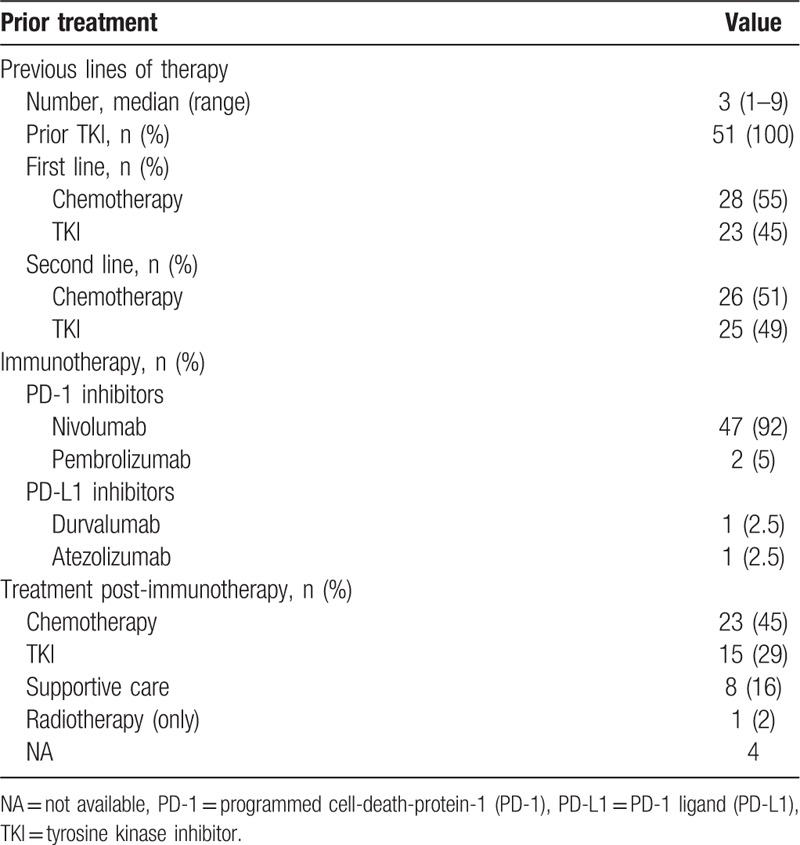
Characteristics of the 51 patients’ prior treatments and immunotherapy.

### 3.2 ICI therapy and clinical outcomes

At immunotherapy initiation, ECOG PS was <2 for 84% (43/51) of the patients (Table 1). Immunotherapy treatments were mainly PD-1 inhibitors: nivolumab for 92% (47/51) of patients and pembrolizumab for 5% (2/51). Seven (13.7%) patients were treated for >9 months with ICI. Post-immunotherapy, 23/51 (45%) patients received chemotherapy and 15/51 (29%) received a TKI (Table 2).

Partial responses (RECIST criteria) were observed in 10 (20%) patients, stable disease in 9 (18%), and progressive disease in 32 (63%). Among the 10 responders, 8 had an *EGFR* mutation and 2 had an *ALK* translocation. Patient characteristics according to type of response are reported in Table 3. The DORs of the *EGFR-*mutated and *ALK*-translocated patients with partial responses were 11.9 (95% confidence interval [CI], 5.6–not reached) months and 9 months (95% CI, 10.9–NR), respectively.

**Table 3:**
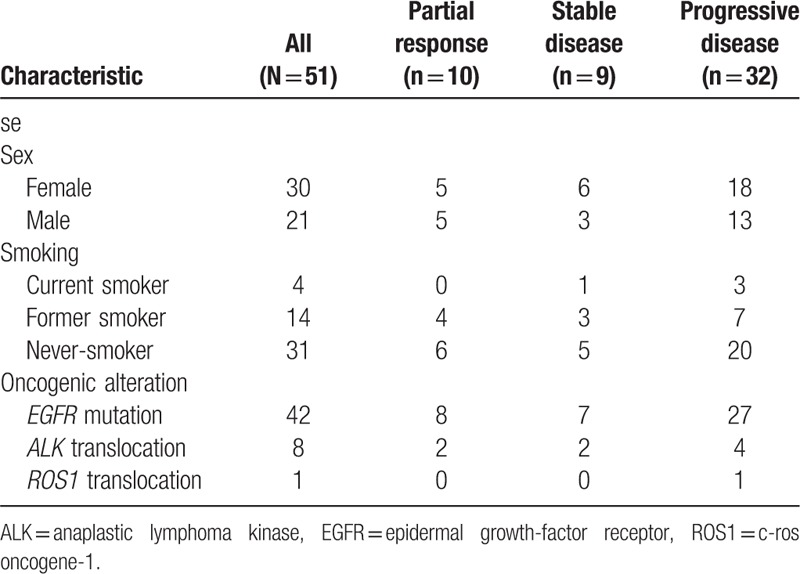
Characteristics of the population according to treatment response.

**Figure 1:**
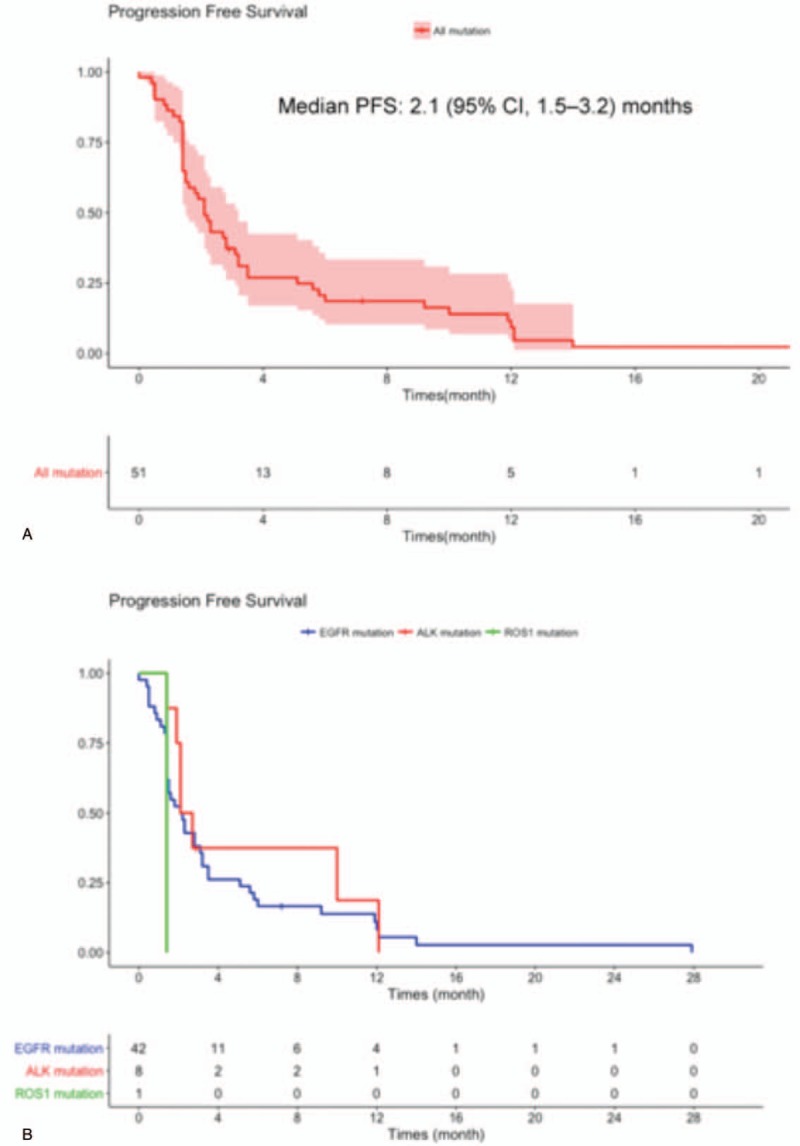
Progression-free survival (PFS) from immunotherapy initiation for the entire cohort (A) and according to the type of molecular alteration (B).

Median follow-up lasted 22 months. Median PFS for the cohort was 2.1 (95% CI, 1.5–3.2) months, with no significant difference (*P* = 0.5) according to the oncogenic mutations: 2.2 (95% CI, 1.4–3.2) months for *EGFR*-mutated patients, 2.4 (95% CI, 2.1–not reached) months for *ALK*-translocated patients and 1.4 months for the *ROS1* patient (Table 4 and Fig. 1). For this cohort, the 12-month PFS rate was 9% (95% CI, 0.03–0.23) and 12-month OS was 63% (95% CI, 0.51–0.78).

**Table 4:**
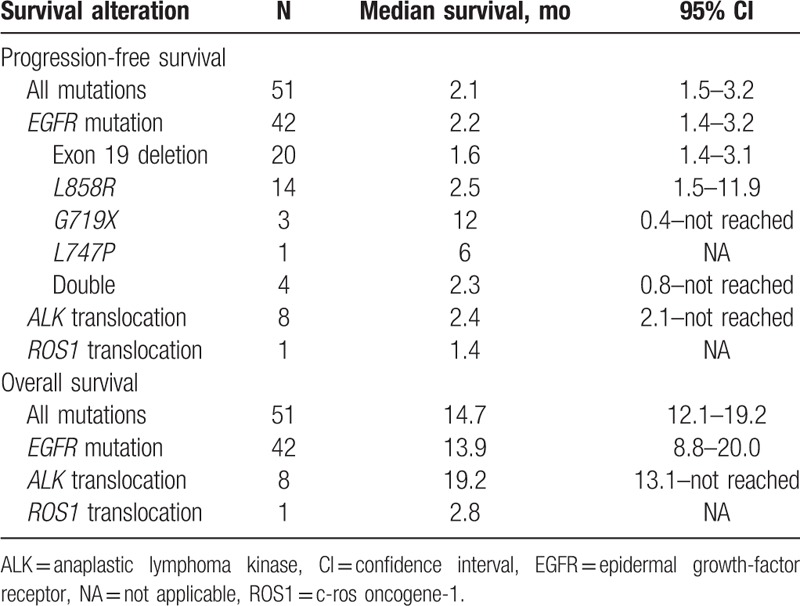
Progression-free survival and overall survival from immunotherapy initiation according to type of molecular alteration.

**Figure 2:**
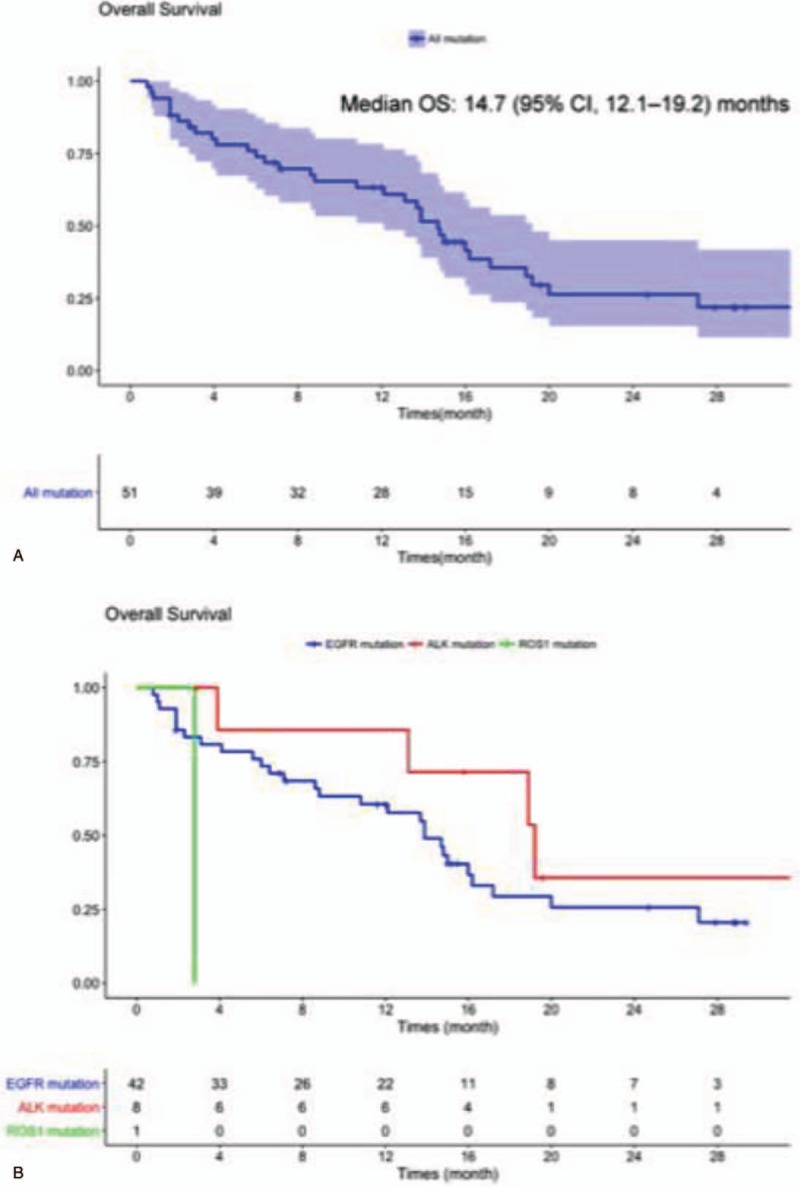
Overall survival (OS) from immunotherapy initiation for the entire cohort (A) and according to the type of molecular alteration (B).

Median OS for the cohort lasted 14.7 (95% CI, 12.1–19.2) months: 13.9 (95% CI, 8.8–20.0) months for *EGFR*-mutated patients, 19.2 (95% CI, 13.1–not reached) months for *ALK*-translocated patients, and 2.8 months for the *ROS1*-translocated patient (Table 4 and Fig. 2).

### 3.3 Safety

Eleven (22%) patients experienced AEs, including 4 (8%) grade 3 to 5 (Table 5). Grade 3 to 5 immune-mediated AEs occurred in 2 patients (hyperthyroidism or hypothyroidism).

**Table 5:**
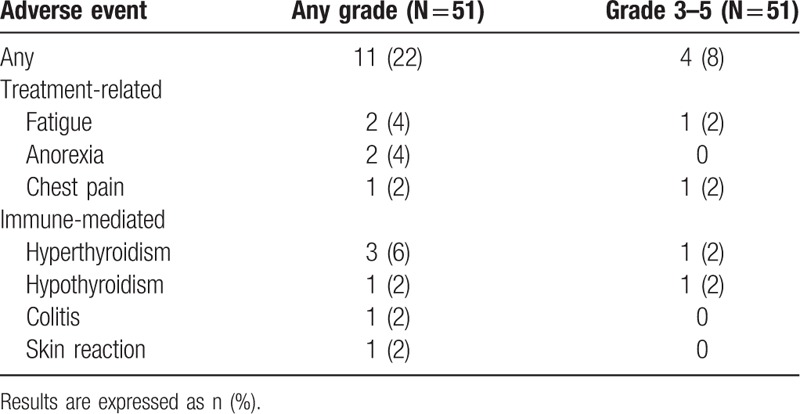
Adverse events on immunotherapy.

## 4 Discussion

This retrospective study included patients with NSCLCs harboring *EGFR*-activating mutations, or *ALK-* or *ROS1*-translocations treated with ICI, after having progressed on targeted treatment and chemotherapy. Their characteristics at enrollment were as expected for a cohort of NSCLC patients including: a high percentage with *EGFR* mutations, 59% women and 61% were never-smokers.^[29]^

Median cohort PFS lasted 2.1 (95% CI, 1.5–3.2) months, with no significant difference according to tumor genotyping. Median OS lasted 14.7 (95% CI, 12.1–19.2) months, with a trend for longer OS for patients with *ALK* mutations (19.2 months).

Only low percentages of patients with *EGFR* mutation (7% –15%) or *ALK* translocation (<1%–4%) had been included in phase III trials on ICI for NSCLC.^[30–32]^ Subgroup analyses of survival data concerned patients with *EGFR* mutations, but not *ALK* translocation because of their small numbers. In the Checkmate-057 study,^[20]^ the OS hazard ratio (HR) for nivolumab versus docetaxel for the subgroup of patients with *EGFR* mutations was 1.18 (95% CI, 0.69–2.00), indicating no benefit of the ICI treatment over chemotherapy. In the Keynote-010 study^[22]^ on NSCLC patients who progressed on platinum therapy, no significant OS benefit was found for ICI treatment in the subgroup of patients with *EGFR* mutations. The OAK study on NSCLC patients with second-line treatment or patients with *EGFR* mutations found that atezolizumab did not prolong OS.^[24]^ Therefore, the median OS observed in our cohort (14.7 months) of heavily pretreated patients was close to that observed for other pretreated and unselected NSCLC patients enrolled in phase III trials.

A meta-analysis of 3 randomized studies with nivolumab, pembrolizumab, or atezolizumab as second-line therapy for advanced NSCLC confirmed that ICI significantly prolonged OS compared with docetaxel for *EGFR* wild-type patients (n = 1362; HR, 0.66 [95% CI, 0.58–0.76]; *P* < .0001), but not *EGFR*-mutated patients (n = 186; HR, 1.05; 95% CI, 0.70–1.55; *P* < .81; treatment–mutation interaction, *P* = .03).^[26]^ Nevertheless, these results must be interpreted prudently because these analyses were computed a posteriori on subgroups with very small samples and without prior stratification on *EGFR*-mutation presence or absence. Moreover, the *EGFR* status was not determined for 19% of patients. In order to properly evaluate PD-1/PD-L1–inhibitor efficacy in *EGFR*-mutated and *ALK*-translocated NSCLC patients, prospective trials specifically enrolling patients with these profiles are needed. The recent ATLANTIC phase II study compared the clinical efficacy of durvalumab as third-line or more for *EGFR–/ALK*– or *EGFR+/ALK+* NSCLC patients according to PD-L1 expression on tumor cells.^[33]^ Patients with *EGFR–/ALK*– NSCLCs had a higher ORR than those with *EGFR+/ALK+* NSCLCs. Nevertheless, their findings suggest that *EGFR+/ALK+* NSCLC patients could also benefit from ICI, especially *EGFR+* patients with ≥25% PD-L1-expressing tumor cells.

Few data obtained in real-life settings from patients with *EGFR* mutations or *ALK* translocations and treated with ICI are available. Gainor et al^[34]^ retrospectively studied 58 NSCLC patients treated with ICI (monotherapy or in combination with EGFR-TKI or chemotherapy): 22 patients with *EGFR* mutations, 6 with *ALK* translocations, and 30 without molecular alterations. Only 1 (3.6%) mutation/translocation-group patient responded, compared with 22.3% of those without molecular alterations. PFS lasted 2.1 (95% CI, 1.8–2.1) months for patients with *EGFR* mutation or *ALK* translocation and 2.6 (95% CI, 1.9–6.4) months (*P* = .018) for those with wild-type *EGFR* and without *ALK* translocation. Although we observed higher response rates herein, PFS (2.1 months) for our cohort was comparable to theirs. Immunotherapy efficacy is very uncertain, particularly as first-line therapy for patients with *EGFR* mutations. In a phase II trial, *EGFR+* patients with TKI-naive PD-L1+ (>1%) expression received first-line pembrolizumab; none responded.^[35]^ The study was stopped due to lack of efficacy.

Another real-life study, a retrospective, multicenter analysis,^[36]^ included 110 *EGFR*-mutated and 18 *ALK*-translocated advanced NSCLC patients treated with ICI. Their median PFS of 2.0 months for *EGFR*-mutated patients and 2.1 months for *ALK*-translocated patients, with median OS at 8.8 and 17 months, respectively, agree with our results.

The relationship between PD-L1 expression and PD-1/PD-L1-inhibitor efficacy against *EGFR*-mutated NSCLCs is controversial. Early retrospective studies reported increased PD-L1 expression in *EGFR*-mutated NSCLCs.^[30–32]^ Notably, Azuma et al^[30]^ reported PD-L1 overexpression in patients with surgically resected NSCLCs harboring *EGFR*-mutations. Those observations seemed to suggest that these patients should be more sensitive to ICIs. However, based on their recent meta-analysis of 18 studies (3969 patients), Soo's et al^[37]^ reported that NSCLCs with *EGFR* mutations were less frequently PD-L1–positive, in comparison to wild-type *EGFR* NSCLCs (HR, 0.59 [95% CI, 0.39–0.92] *P* < .021). That meta-analysis highlighted the marked heterogeneity among the studies in the absence of standardized methods to determine PD-L1 expression.

Several mechanisms potentially explaining the poor response of pretreated *EGFR*-mutant NSCLCs to PD-1/PD-L1 inhibitors have been proposed. Among them, a lack of T-cell infiltration into the tumor microenvironment could explain lower responses to PD-1/PD-L1–pathway blockade.^[38]^ Ongoing clinical trials have been designed to combine ICI and TKI as a strategy for optimizing their efficacies in patients with *EGFR*-mutated or *ALK-*translocated NSCLCs.^[27]^ The phase III randomized IMpower-150 trial compared patients with stage-IV non-squamous NSCLCs, ECOG PS = 0/1, with 3 arms: carboplatin–paclitaxel–bevacizumab, atezolizumab–platinum-based chemotherapy + bevacizumab (quadritherapy) or without.^[39]^ Patients received 4 to 6 treatment cycles and maintenance therapy with bevacizumab, atezolizumab + bevacizumab, or atezolizumab, depending on the arm, until progression. Quadritherapy, compared with carboplatin–paclitaxel–bevacizumab, respectively, obtained a significant PFS benefit (8.3 vs 6.8 months), and an OS gain (19.8 vs 14.9 months).^[38]^ That benefit was observed regardless of the tumor cell or inflammatory cell (IC) PD-L1–expression level, even when those cells were PD-L1–negative and was even better for patients with liver metastases. An important element was the notable quadritherapy efficacy for patients with *EGFR* mutations or *ALK* translocations with disease progression after targeted therapy. Median OS was not reached (NR vs 17.5 months; HR 0.54 [95% CI 0.29–1.03]).^[39]^

When efficacy was observed in this real-life study on oncogenically mutated NSCLCs, PFS and OS were always close to those obtained by patients without such genetic anomalies.^[40,41]^ Thus, for 303 non-selected patients with advanced NSCLCs progressing after a platinum-doublet chemotherapy, median PFS and OS on nivolumab were 2.6 (95% CI 2.1–3.5) and 11.3 (95% CI: 8.5–13.8) months, respectively, similar to the 2.4 and 14.7 months reported herein. In another recent analysis on 530 patients evaluated for *KRAS* mutations, 206 (39%) were positive while 324 (61%) carried wild-type *KRAS*. *KRAS* status did not influence nivolumab efficacy in terms of ORR (20% vs 17%, *P* = .39) and disease control rate (47% vs 41%, *P* = .23). For the *KRAS*-positive/mutated and *KRAS*-negative/wild-type groups, respectively, median PFS lasted 4 and 3 months, and median OS 11.2 and 10 months. As in our study, observed PFS is disappointing especially considering 20% RR.^[40]^

Grade 3–5 AEs occurred in 8% of the cohort patients. Immune-mediated AEs were expected and the most frequent was hyperthyroidism for 3 patients, including 1 patient with grade 3 to 5. These results obtained in a real-life setting confirm the good ICI safety profile reported in phase III trials.

Our findings do not support decreased efficacy of PD-1/PD-L1 inhibitors in pretreated patients with an *EGRF* mutation or *ALK* translocation. Some limitations must nevertheless be taken into consideration. It has the limitation inherent in retrospective studies; the analyses rely on data recorded in patient files and, therefore, must be interpreted with caution. PFS, OS, and ORR were not compared for patients harboring *EGFR* mutations or *ALK/ROS* translocations and those without. If ICI PFS appeared close to that observed in pretreated unselected NSCLC patients in randomized-controlled trials the more promising OS probably linked to post ICI treatments. Clinical outcomes according to PD-1 expression were not reported because this evaluation was rarely done routinely at the onset of the management of these patients. PD-L1 expression of could not be obtained for the majority of patients because it simply was not part of the diagnostic work-up of patients in 2014 to 2015 and, by the time it became standard practice, most of the tumor material had most often already been exhausted. Finally, in light of the retrospective design of the study, AEs were probably underestimated, especially grade 1/2. Nonetheless, one of the study's strengths is the enrollment of a real-life cohort composed of 51 heavily pretreated patients with molecular alterations given ICI inhibitors, a rare patient profile in randomized-clinical trials.

## 5 Conclusion

In this real-world setting analysis, ICI PFS in *EGFR*-mutated, *ALK-* or *ROS1*-translocated NSCLC patients appeared close to that observed in pretreated unselected NSCLC patients in randomized-controlled trials or observational studies. The more promising OS probably linked to post ICI treatments. Large prospective studies on these patient subsets are needed to better discern the place of ICIs in their treatment.

## Author contributions

**Conceptualization:** Olivier Bylicki, Florian Guisier, Maurice Perol, Christos Chouaid.

**Data curation:** Olivier Bylicki, Florian Guisier, Hélène Doubre, Pierre Fournel, Régine Lamy, Jean-Bernard Auliac.

**Formal analysis:** Olivier Bylicki, Henri Janicot, Maurice Perol, Christos Chouaid.

**Funding acquisition:** Isabelle Monnet, Radj Gervais, Pierre Fournel, Jean-Bernard Auliac, Christos Chouaid.

**Investigation:** Olivier Bylicki, Isabelle Monnet, Hélène Doubre, Radj Gervais, Henri Janicot, Maurice Perol, Pierre Fournel, Régine Lamy, Jean-Bernard Auliac.

**Methodology:** Olivier Bylicki, Florian Guisier, Isabelle Monnet.

**Project administration:** Olivier Bylicki, Hélène Doubre, Jean-Bernard Auliac, Christos Chouaid.

**Resources:** Jean-Bernard Auliac.

**Supervision:** Olivier Bylicki.

**Validation:** Olivier Bylicki, Isabelle Monnet, Hélène Doubre, Radj Gervais, Henri Janicot, Maurice Perol, Pierre Fournel, Régine Lamy, Jean-Bernard Auliac, Christos Chouaid.

**Writing – original draft:** Olivier Bylicki.

**Writing – review & editing:** Olivier Bylicki, Christos Chouaid.

Christos Chouaid orcid: 0000-0002-4290-5524.
